# Supplementary material for: Unlocking a high bacterial diversity in the coralloid root microbiome from the cycad genus Dioon
Source: PLoS One. 2019 Feb 6;14(2):e0211271. doi: 10.1371/journal.pone.0211271 (PMC6364921; doi:10.1371/journal.pone.0211271)
Supplement: S1 Table — (DOCX) [file pone.0211271.s001.docx]

| **Sample** | **Observed species** | **Shannon effective** | **Simpson effective** |
| --- | --- | --- | --- |
| 01.DSO | 1593 | 538.04 | 160.04 |
| 04.DME | 1267 | 349.68 | 105.06 |
| 01.DPU | 1030 | 198.56 | 48.05 |
| 01.DAN | 793 | 147.79 | 36.17 |
| 03.DME | 718 | 139.84 | 42.53 |
| 02.DSO | 691 | 132.94 | 40.36 |
| 05.DME | 671 | 29.13 | 4.2 |
| 01.DED | 559 | 110.02 | 44.08 |
| 01.DME | 557 | 83.02 | 23.35 |
| 02.DME | 555 | 78.78 | 21.7 |
| 06.DME | 428 | 65.65 | 16.32 |
| 01.DSP | 419 | 13.88 | 3.21 |

S1. Table
