# Supplementary material for: Unlocking a high bacterial diversity in the coralloid root microbiome from the cycad genus Dioon
Source: PLoS One. 2019 Feb 6;14(2):e0211271. doi: 10.1371/journal.pone.0211271 (PMC6364921; doi:10.1371/journal.pone.0211271)
Supplement: S2 Table — (DOCX) [file pone.0211271.s002.docx]

**S2. Table**

|  |  |  | | **Alfa diversity (p value)** | | |  |
| --- | --- | --- | --- | --- | --- | --- | --- |
| **Comparison** | **Compartment** | | **Population** | **Observed species** | **Shannon effective** | **Simpson effective** |  |
| **Set “Natural population” versus *Dioon merolae* samples from the botanical garden** | END | | NAP vs BOG | 0.61 | 0.35 | 0.26 |  |
| **Set “Rhizosphere” versus their respective endosphere samples** | | RHZ, END | | BOG | 0.004** | 0.002** | 0.026 |
| **Set “Bulk soil” versus their respective rhizosphere and endosphere samples** | BSO, RHZ, END | | BOG | 0.04^NS^ | 0.04^NS^ | 0.06 |  |

END = Endosphere; RHZ = Rhizosphere; BSO = Bulk soil; NAP = Natural population; BOG = Botanical garden. NS= Not significant after test paired with Bonferroni correction; ** p<0.01
