# Supplementary figures and images for: Unlocking a high bacterial diversity in the coralloid root microbiome from the cycad genus Dioon
Source: PLoS One. 2019 Feb 6;14(2):e0211271. doi: 10.1371/journal.pone.0211271 (PMC6364921; doi:10.1371/journal.pone.0211271)

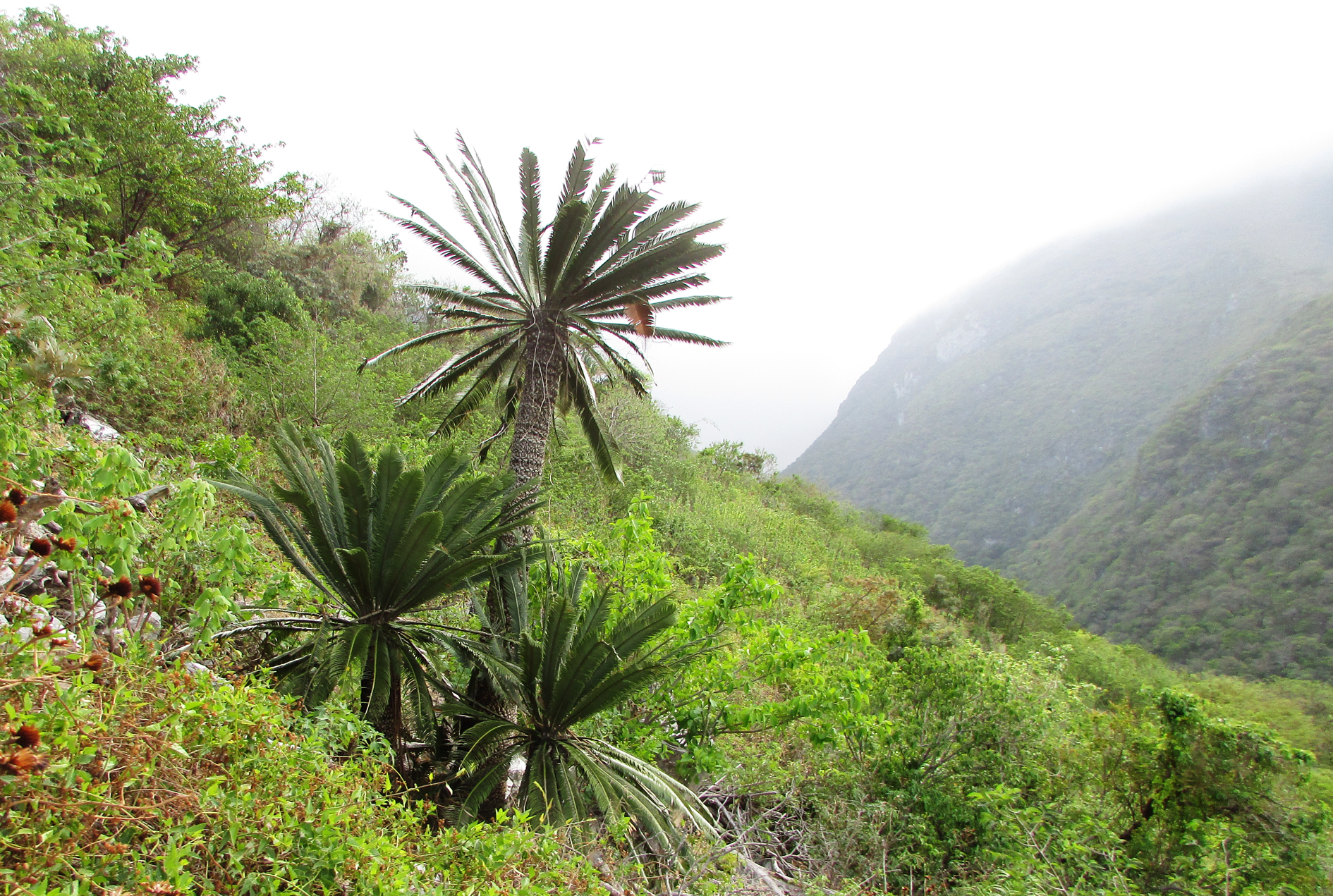

Supplement: S1 Fig — (TIF) [file pone.0211271.s003.tif]

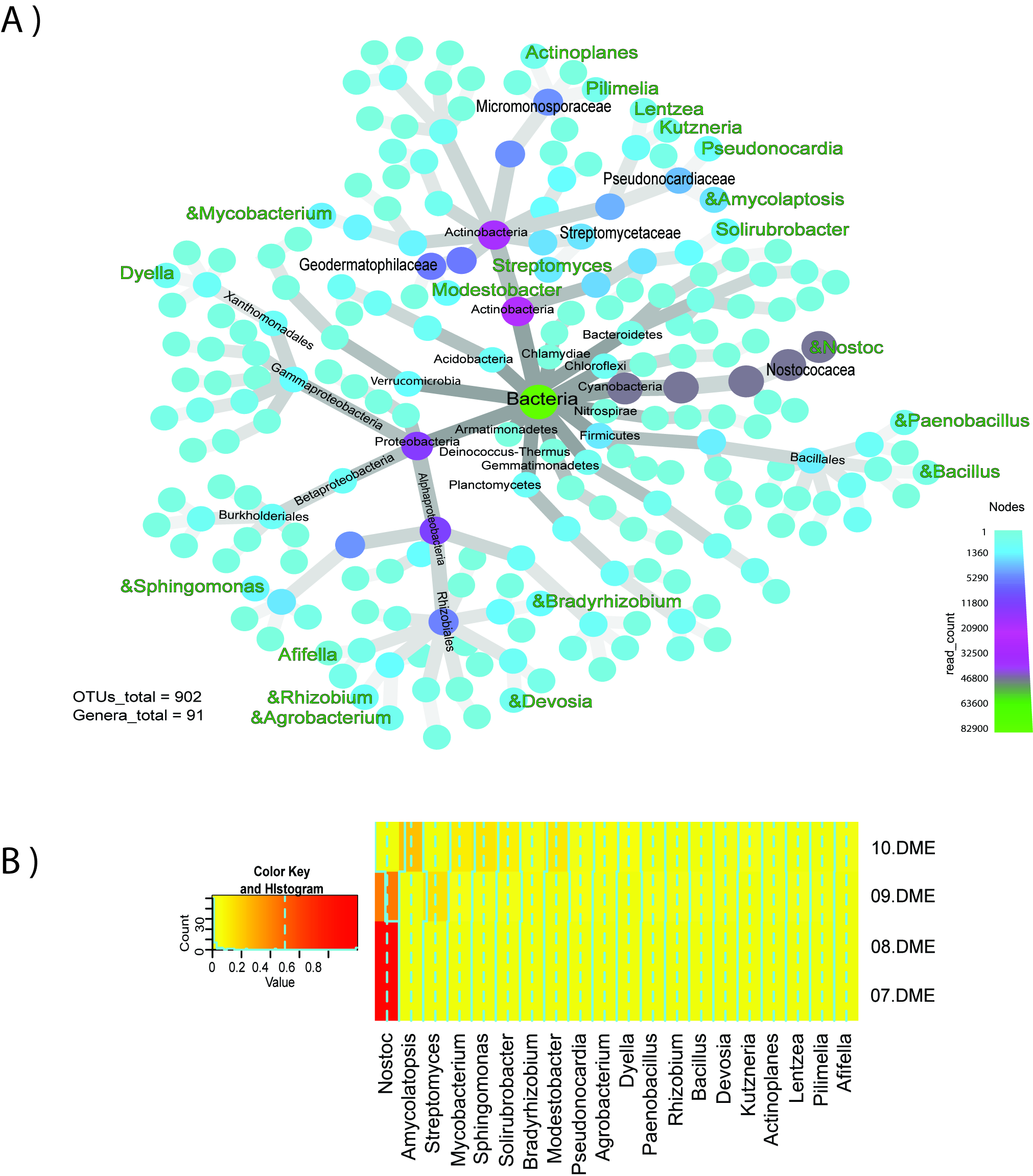

Supplement: S2 Fig — A) Heat tree of taxonomic diversity, the node width and color indicate the number of reads assigned to each taxon. Of the 20 most abundant genera (green) the symbol “&” represents the bacteria that have been reported as nitrogen-fixing. B) Heat map of 20 most abundant genera, each column corresponds to a bacterial genus, each row to a specific Dioon merolae sample. (TIF) [file pone.0211271.s004.tif]

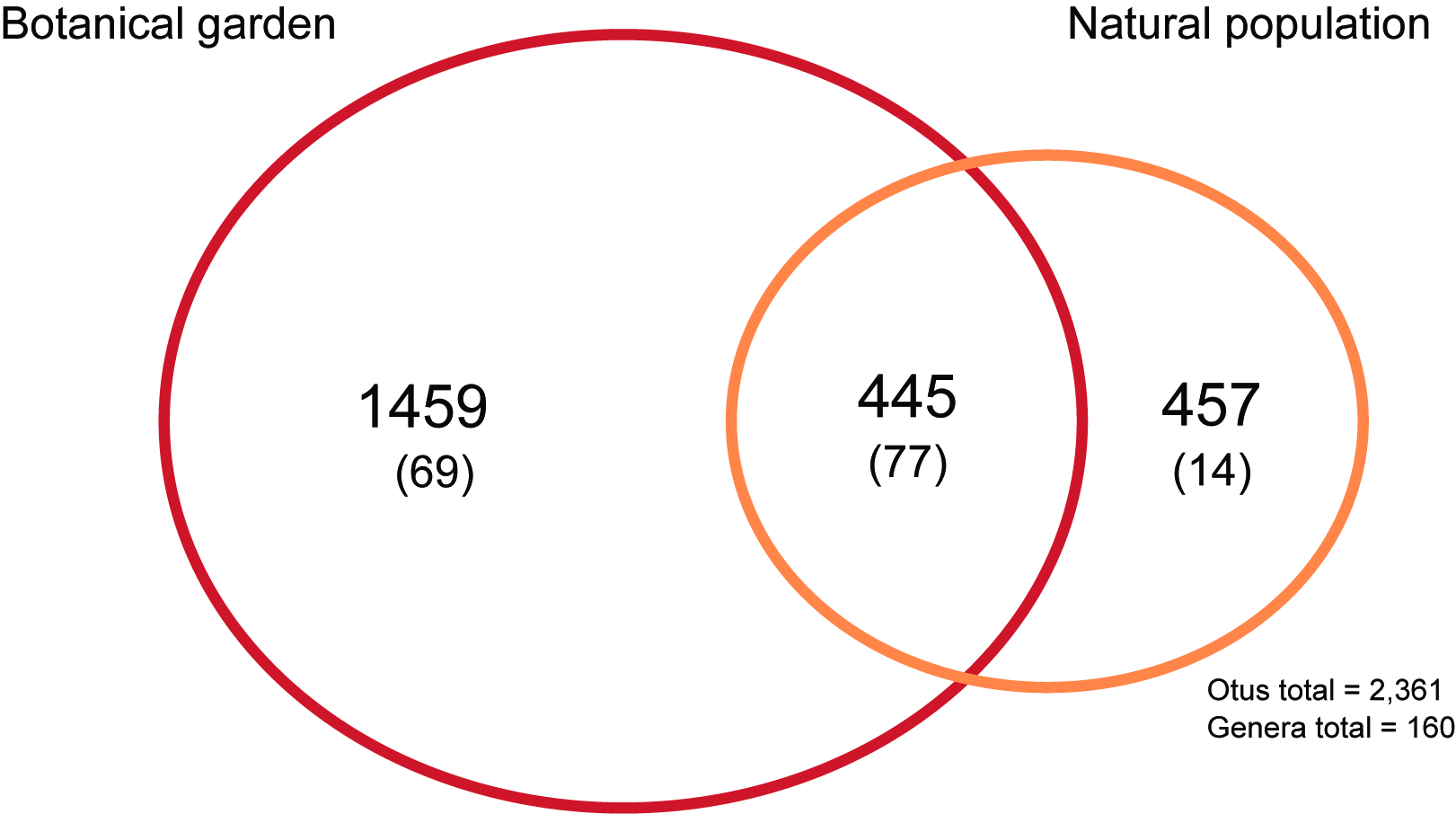

Supplement: S3 Fig — Venn diagram showing the shared OTUs and genera (in parenthesis) between the D. merolae samples from botanical garden and natural population. (TIF) [file pone.0211271.s005.tif]
